# Trends in Supply of Nursing Home Beds, 2011-2019

**DOI:** 10.1001/jamanetworkopen.2023.0640

**Published:** 2023-03-01

**Authors:** Katherine E. M. Miller, Paula Chatterjee, Rachel M. Werner

**Affiliations:** 1 Division of Medical Ethics and Health Policy, Perelman School of Medicine at the University of Pennsylvania, Philadelphia; 2 Leonard Davis Institute of Health Economics, University of Pennsylvania, Philadelphia; 3 General Internal Medicine, Perelman School of Medicine, Philadelphia, Pennsylvania; 4 Center for Health Equity Research and Promotion, Crescenz VA Medical Center, Philadelphia, Pennsylvania

## Abstract

**Importance:**

Nursing homes play a vital role in providing postacute and long-term care for individuals whose needs cannot be met in the home or community. Whether the supply of nursing home beds and, specifically, the supply of high-quality beds has kept pace with the growth of the older adult population is unknown.

**Objective:**

To describe changes in the supply of population-adjusted nursing home beds from 2011 to 2019.

**Design, Setting, and Participants:**

This cross-sectional study examines changes in the population-adjusted supply of nursing home beds across all US counties from 2011 to 2019 and describes county and nursing home characteristics where the supply of nursing home beds has increased vs decreased.

**Main Outcomes and Measures:**

Number of nursing home beds adjusted per 10 000 adults aged 65 years and older.

**Results:**

The population-adjusted supply of nursing home beds declined from 2011 to 2019 for 86.4% of US counties, by a mean (SD) of 129.9 (123.8) beds per 10 000 adults aged 65 years or older per county from a baseline mean (SD) of 552.5 (274.4) beds per 10 000 adults aged 65 years or older per county in 2011. The share of beds that were high quality (4- or 5-star ratings) also declined, which was driven by a small number of counties where nursing home bed supply increased due to a proliferation of lower-quality beds. Simultaneously, metropolitan counties with declining numbers of nursing home beds also experienced declining number of senior housing residential beds (−11.3 [54.6] beds per 10 000 adults aged 65 years or older per county from a baseline mean [SD] of 354.8 [222.3]).

**Conclusions and Relevance:**

The findings of this cross-sectional study suggest that the supply of nursing home beds, specifically high-quality nursing home beds, and senior residential housing beds have not kept pace with the demographics of an aging population. Understanding the supply of high-quality nursing home beds and associated geographic variation can inform targeted policies to best support older adults requiring nursing home care.

## Key Points

**Question:**

Is the supply of nursing home beds, specifically, high-quality beds, adequate for the aging population in the US?

**Findings:**

In this cross-sectional study, the supply of nursing home beds per 10 000 adults aged 65 years or older per county declined from 2011 to 2019 in 86.4% of US counties, by a mean of 129.9 beds from 552.5 beds in 2011. The share of 4- and 5- star beds also decreased, likely driven by an increasing number of low-quality beds where bed supply increased.

**Meaning:**

These findings suggest declining availability of nursing home beds, particularly high-quality beds, raising concerns about whether the long-term care needs of an aging population are being met.

## Teaser

This cross-sectional study examines changes in the supply of population-adjusted nursing home beds from 2011 to 2019 in the US.

## Introduction

Nursing homes play a vital role by providing postacute and long-term care for individuals whose needs cannot be met in the community due to care complexity or limited resources.^1^ However, there are growing concerns that the supply of nursing home beds may be declining faster than the demand for them warrants,^2^ a concern that has grown with the increase in nursing homes closures during the pandemic.^3^ The supply of nursing home beds may be declining, in part, because of individual preferences to remain in the community for as long as possible rather than receiving care in a nursing home.^4^ It may also be declining in the context of increasing availability of home- and community-based care.^5,6^ However, as the US population has aged and required more assistance with care,^7^ it is unclear whether the supply of nursing home beds has kept pace with demand.

In addition to ensuring an adequate supply of nursing home beds, ensuring access to high-quality nursing home care has the potential to improve patient outcomes. Patients residing in better quality nursing homes have lower rates of hospitalizations and mortality compared with residents at nursing homes of worse quality.^1,8,9^ If the supply of high-quality nursing home beds is declining, this may have adverse consequences for nursing home residents. Understanding trends in the supply of nursing home beds and, in particular, the supply of high-quality nursing home beds is crucial for policymakers seeking to better align access to high-quality nursing home care with the needs of an aging population. However, whether the supply of high-quality nursing home beds has changed over time is unknown.

In this study, we describe changes in the supply of US nursing home beds from 2011 to 2019 after adjusting for changes in the number of older adults in the population. We next identify and describe characteristics of counties where the supply of nursing home beds has increased vs decreased over this period, including the supply senior housing beds, which are potential alternative residential long-term service and supports, to evaluate whether changes in seniors’ housing may have offset changes in the availability of nursing home beds. Finally, we identify and describe characteristics of the nursing homes beds in counties where the supply of nursing home beds has increased vs decreased throughout the same period, including quality of care.

## Methods

This cross-sectional study followed the Strengthening the Reporting of Observational Studies in Epidemiology (STROBE) reporting guideline. The institutional review board at the University of Pennsylvania deemed this study exempt because of the use of publicly available facility-level data. We used a serial cross-sectional study design to examine supply of nursing home beds at the county-level from 2011 to 2019.

### Data and Sample

We used 5 primary data sources to create a county-year–level data set. First, we used the LTCFocus data set to identify the number of nursing home beds or supply in all US nursing homes (including both skilled nursing facility and long-term care beds) and nursing home characteristics (for-profit status, membership in a chain, percent of nursing home residents whose primary support is Medicaid). LTCFocus is a national data set of nursing home facilities derived from the Minimum Data Set to yield patient characteristics, case-mix, and staffing measures aggregated to the facility, county, and state levels. LTCFocus is sponsored by the National Institute on Aging through a cooperative agreement with the Brown University School of Public Health.^10^

Second, we use the Centers for Medicare & Medicaid Services’ Nursing Home Compare data set to measure nursing home quality using overall 5-star ratings for each year.^11^ We identify higher quality nursing homes as those with 4- or 5-star ratings, indicating higher quality care.^8,9^ We use the threshold of 4- or 5-star ratings as informed by past studies.^12,13,14,15^ For all nursing home characteristics, we calculated the number of beds in a given nursing home type in each county-year (eg, the number of for-profit nursing home beds per county-year).

Third, we used the Area Health Resource File over the same period to identify county-year–level characteristics, including 2013 Rural Urban Continuum Codes (1-3, metropolitan; 4-6, nonmetropolitan and adjacent to metropolitan area; 7-9, nonmetropolitan and nonadjacent to metropolitan area), percent of Medicare beneficiaries dually eligible for Medicaid, mean Hierarchical Chronic Condition (HCC) scores for Medicare Fee-for-Service (FFS) beneficiaries (a clinical risk score), and the percent of beneficiaries enrolled in Medicare Advantage. The Area Health Resource File is produced by the Health Resources and Services Administration and provides county-level data aggregated from more than 60 sources to provide data including, but not limited to, the areas of health workforce, health facilities, population demographics, and population economic characteristics.^16^

Fourth, we used data from the American Community Survey 5-year estimates to identify additional population-level characteristics in each county-year, including the percent of the population aged 65 years or older, median household income, and the percent of adults 65 years or older living in poverty. Fifth, we used NIC MAP, provided by NIC Analytics at National Investment Center for Seniors Housing and Care to obtain information on senior housing communities (ie, assisted living facilities, continuing care retirement communities, and independent living communities). We calculated the number of senior housing residential beds in a county-year, including bed counts in the 140 largest metropolitan areas in the US for 2015 to 2019, the only years and geographic areas for which this survey was available.^17^

### Statistical Analysis

We described trends in the number of nursing home beds per county-year per 10 000 adults aged 65 years or older from 2011 to 2019. Then, we categorized counties into 3 groups according to their change in nursing home bed supply per 10 000 adults aged 65 years or older between 2011 and 2019: (1) increase in number of nursing home beds, (2) decrease in number of nursing home beds, or (3) no change. We defined no change as counties with an increase or decrease up to 10 beds per 10 000 adults aged 65 years or older from 2011 to 2019 (eFigure in Supplement 1 ).

Next, we described characteristics of counties and nursing home beds overall and across these 3 categories of change in nursing home bed supply. We conducted multiple sensitivity analyses. First, we extended to study period to include 2020, examining trends in nursing home bed supply from 2011 to 2020 to identify changing trends during the first year in the COVID-19 pandemic. We retained the period of 2011 to 2019 as the primary analysis given that our focus was on changes over time, not changes specific to the pandemic. Second, as identifying an appropriate threshold for no meaningful change in the number of nursing home beds in a county is arbitrary, we repeated our analyses using a threshold of a change of 2.5% in the number of nursing home beds in a county rather than the 10-bed threshold we use in the primary analysis. We conducted all analyses in StataMP version 17 (StataCorp).

## Results

We included 15 564 nursing homes across 2916 US counties, accounting for 1 661 276 nursing home beds in 2011. At a county-level, there were a mean (SD) of 558.7 (293.6) beds per 10 000 adults aged 65 years or older in 2011.

**Figure 1.**
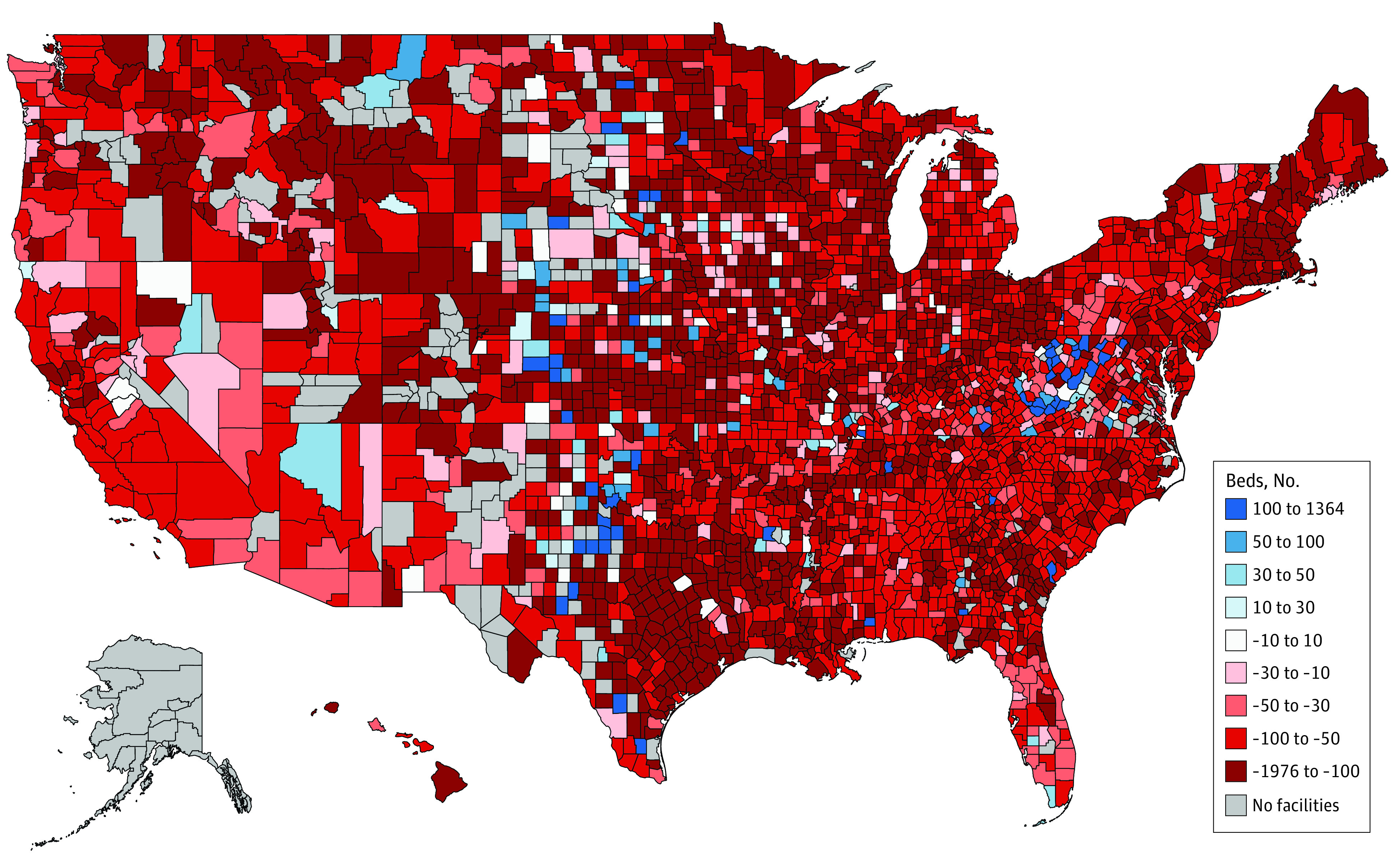
Change in Number of Nursing Home Beds per 10 000 Adults Aged 65 Years or Older, 2011-2019 Data for the number of nursing home beds per county per year are from LTCFocus.^10^ Data for the number of adults aged 65 years or older per county per year are from Social Explorer, which are derived from the American Community Survey 5-year estimates.^18,19^

**Figure 2.**
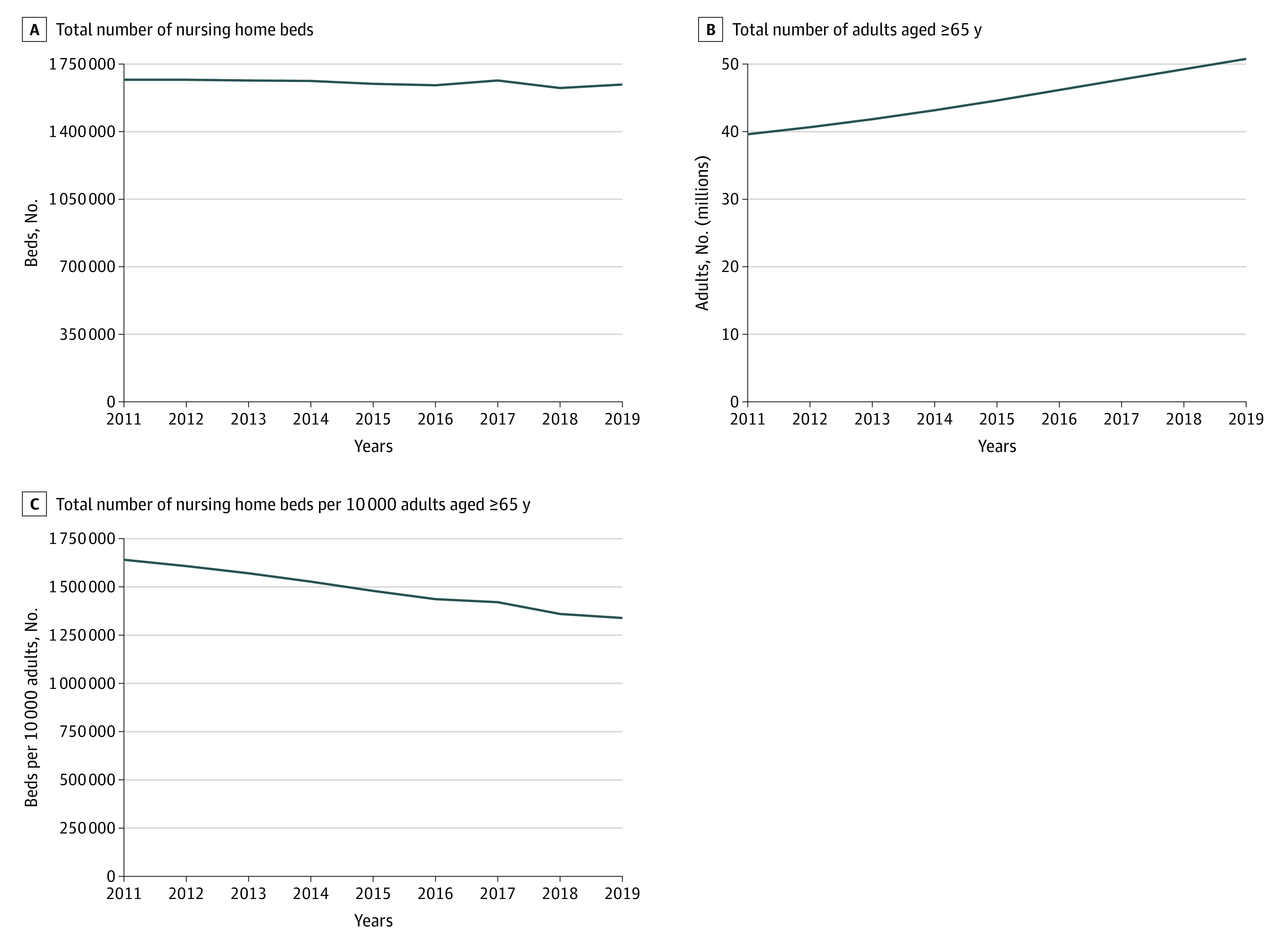
Trends in Number of Nursing Home Beds, Population Aged 65 Years or Older, and Nursing Home Beds per Population Aged 65 Years or Older, 2011-2019 Data for the number of nursing home beds per county per year are from LTCFocus.^10^ Data for the number of adults aged 65 years or older per county per year are from Social Explorer, which are derived from the American Community Survey 5-year estimates.^18,19^

From 2011 to 2019, most counties experienced a decline in population-adjusted supply of nursing home beds (Figure 1).^10,18,19^ The mean (SD) number of population-adjusted county-level nursing home beds decreased by 113.5 (141.6) beds per 10 000 adults aged 65 years or older between 2011 and 2019, from 558.7 (293.6) to 445.2 (281.9) beds per 10 000 adults aged 65 years or older (a relative decrease of 20.3%). In 2720 of 2916 counties (86.5%), the population-adjusted supply decreased; it increased in 147 counties (4.7%); and there was no change in 9 counties (1.6%) (Table 1). The decrease in population-adjusted number of nursing home beds was driven by both a decrease in the absolute number of nursing home beds and an increase in the population aged 65 years or older (Figure 2).^10,18,19^ The increase in population was more pronounced than the decrease in beds (Figure 2).^10,18,19^ From 2011 to 2019, the absolute number of beds decreased by 1.5% (from 1 668 761 beds to 1 644 034 beds while the population of adults aged 65 years or older increased by 28.2% (from 39 607 672 adults to 50 783 796 adults).

**Table 1.** County Characteristics by Increase and Decrease of Nursing Home Beds^a^

Characteristics	Counties with no nursing home in 2011 or 2019 (n = 230)	Counties with ≥ 1 nursing home in 2011 or 2019			
		All (n = 2916)	Decrease (n = 2720)	No change (n = 49)	Increase (n = 147)
**Demographics**					
% Of population					
≥65 years in 2011	15.8 (6.5)	15.7 (4.0)	15.5 (3.9)	18.9 (3.6)	18.6 (4.6)
≥65 living in poverty in 2011	11.3 (8.1)	10.8 (4.8)	10.7 (4.8)	10.9 (4.4)	12.6 (5.3)
Medicare population, mean (SD)					
% Of Medicare beneficiaries eligible for Medicaid in 2011	21.1 (12.7)	22.5 (8.7)	22.5 (8.7)	19.0 (6.8)	23.0 (9.4)
Medicare FFS beneficiaries average HCC score in 2011	0.85 (0.14)	0.9 (0.1)	0.9 (0.1)	0.9 (0.1)	0.9 (0.1)
% Medicare Advantage penetration in 2011	13.0 (8.4)	16.9 (12.1)	17.2 (12.2)	10.1 (8.9)	11.6 (8.6)
Availability of other senior housing, mean (SD)^b^					
No. of residential beds per 10 000 adults aged ≥65 years in 2015	233.6 (145.2)	354.8 (222.3)	354.6 (222.5)	646.0 (NA)^c^	266.4 (45.3)
Change in No. of residential beds per 10 000 adults aged ≥65 years, 2015-2019	54.0 (199.6)	−11.3 (54.6)	−11.9 (53.5)	147.9 (NA)^c^	65.2 (177.3)
**Geographic characteristics**					
Rurality by rural urban continuum code, No. (%)					
Metropolitan (codes 1-3)	43 (19.0)	1123 (38.5)	1098 (40.4)	6 (12.2)	19 (12.9)
Nonmetropolitan or adjacent to metropolitan area (codes 4-6)	17 (7.5)	882 (30.2)	833 (30.6)	14 (28.6)	35 (23.8)
Nonmetropolitan or nonadjacent to metropolitan area (codes 7-9)	166 (73.5)	911 (31.2)	789 (29.0)	29 (59.2)	93 (63.3)
Census region, No. (%)					
Midwest	40 (17.4)	1015 (34.8)	937 (34.4)	26 (53.1)	52 (35.4)
Northeast	4 (1.7)	213 (7.3)	213 (7.8)	0	0
South	79 (34.3)	1343 (46.1)	1244 (45.7)	16 (32.7)	83 (56.5)
West	107 (46.5)	345 (11.8)	326 (12.0)	7 (14.3)	12 (8.2)

Abbreviations: FFS, fee-for-service; HCC, hierarchical condition categories; NA, not available.

^a^ Data sources include demographic and economic characteristics from Social Explorer; Medicare population and geographic characteristics from the Area Health Resource File^16^; and senior housing characteristics are from the National Investment Center for Seniors Housing and Care.^17^ No change is defined as counties with an increase or decrease of 10 beds per 10 000 adults aged 65 years or older from 2011 to 2019. Hierarchical condition categories calculated by Centers for Medicare & Medicaid Services.

^b^ Senior housing and retirement community beds are only available from the 140 largest metropolitan areas for years 2015 to 2019.

^c^ Because of the subsample of senior housing and retirement community beds from the 140 largest metropolitan areas, only 1 county is allocated to the no change category, and therefore, no SD is presented.

Compared with counties with an increase in the population-adjusted supply of nursing home beds, counties with a decrease in supply had a lower baseline percent of population aged 65 years or older (mean [SD], 15.5% [3.9%] vs 18.6% [4.6%]) and a lower baseline percent of adults aged 65 years or older living in poverty (mean [SD], 10.7% [4.8%] vs 12.6% [5.3%]) (Table 1). These counties also had a higher baseline percent of population enrolled in Medicare Advantage (mean [SD], 17.2% [12.2%] vs 11.6% [8.6%]) and were disproportionately in metropolitan counties (mean [SD], 1098 of 2720 [40.4%] vs 19 of 147 [12.9%]). In addition, counties with a decrease in the supply of nursing home beds also had a decrease in the number of population-adjusted senior housing residential beds, while counties with an increase in the supply of nursing home beds had an increase in the number of senior housing residential care beds (mean [SD], −11.9 [53.5] vs 65.2 [177.3]).

Although the overall population-adjusted supply of nursing home beds declined, these declines were not distributed evenly across nursing home bed characteristics. Overall, there was a small increase in the share of for-profit beds and a small decrease in the share of beds that were 4- or 5-star beds between 2011 and 2019 (<1 percentage point) (Table 2). In counties where the supply of nursing home beds declined, the percentage of beds that were for-profit or 4- or 5-star rating increased by less than 1 percentage point over the study period. However, in counties where the supply of nursing home beds increased, the share of beds that were for-profit grew by 2.3 percentage points and the share of beds that were 4- or 5-star declined by 5.5 percentage points in these counties.

**Table 2.** Nursing Home Bed Characteristics Across Counties Categorized by Increasing and Decreasing of Nursing Home Beds, 2011-2019^a^

Characteristics	All	Decreasing	No change	Increasing
Counties, No. (%)	2916	2720 (93.3)	49 (1.7)	147 (5.0)
No. of beds per 10 000 adults aged ≥65 years per county in 2011, mean (SD)	558.7 (293.6)	552.5 (274.4)	652.4 (323.4)	640.6 (523.2)
Change in No. of beds per 10 000 adults aged ≥65 years 2011 to 2019	−113.5 (141.6)	−129.9 (123.8)	−1.3 (6.0)	151.2 (188.1)
% Of for-profit beds in 2011, mean (SD)^b^	65.6 (36.8)	66.5 (36.1)	52.7 (43.6)	51.3 (45.1)
% Point change in for-profit beds 2011 to 2019	0.2 (23.3)	0.2 (22.9)	−1.7 (29.8)	2.3 (30.1)
% Of chain-affiliated beds in 2011, mean (SD)^b^	55.1 (37.1)	55.7 (36.6)	45.8 (41.9)	45.5 (44.2)
% Point change in chain-affiliated beds 2011 to 2019	1.8 (33.5)	1.7 (33.0)	10.0 (29.6)	0.9 (43.7)
% Of 4- or 5-star beds in 2011, mean (SD)^b^	40.6 (36.2)	40.2 (35.7)	51.3 (41.9)	46.6 (44.0)
% Point change in 4- or 5-star beds 2011 to 2019	−0.2 (46.0)	0.1 (45.6)	0.9 (36.9)	−5.5 (56.4)
% Of residents whose primary support is Medicaid in 2011, mean (SD)^c^	62.8 (14.6)	63.5 (13.3)	61.0 (15.1)	51.0 (26.8)
% Point change in percent of residents whose primary support is Medicaid 2011 to 2019	0.1 (16.2)	−0.7 (14.6)	−0.7 (17.5)	15.7 (30.1)

^a^ Calculations of nursing home beds are adjusted for 10 000 adults aged 65 years or older in the nursing home’s county for 2011 to 2019.

^b^ Percent is based on all nursing home beds in a county (eg, the percent of for-profit nursing home beds out of all nursing home beds in a county).

^c^ Percent of residents whose primary support is Medicaid is measured at the nursing home level.

When examining trends from 2011 to 2020 as a sensitivity analysis, we observed a more dramatic decrease in the number of beds per 10 000 adults aged 65 years or older than when examining trends from 2011 to 2019 (158.4 vs 113.5, respectively). Trends in beds across other nursing home characteristics were variable (eTable 1 in Supplement 1). Results of the sensitivity analysis using an alternative threshold of positive or negative 2.5% change in number of beds to indicate meaningful change were also consistent with overall findings (eTable 2 in Supplement 1).

## Discussion

In this cross-sectional study, we found that the population-adjusted supply of nursing home beds has declined over the past decade. The share of high-quality beds (ie, 4- or 5-star ratings) also declined, particularly in counties where nursing home bed supply increased during this time, suggesting that the decline in the share of high-quality beds was driven by a proliferation of lower-quality beds. Additionally, there was a decline in the number of senior housing beds during this time. Understanding how nursing home bed supply has changed over time can inform policies focused on aligning access to high-quality nursing home care with the growing needs of an aging population.

The broad decrease in supply of nursing home beds may reflect state and federal policy initiatives to rebalance long-term services and supports away from institutions and toward home- and community-based settings.^5,6^ For example, the Affordable Care Act offered federal funding to states that spent more than 50% of Medicaid Long-Term Services and Supports funds on home- and community-based services (HCBS) to shift care from institutions into homes. This has contributed to broad expansions of HCBS nationally. However, shortages of direct care workers to provide HCBS, lack of affordable and accessible housing for nursing home residents to receive care in noninstitutional settings, and complex processes to access Medicaid HCBS collectively result in barriers to accessing HCBS, which have been exacerbated during the COVID-19 pandemic.^20,21,22,23^ The availability of HCBS has not yet met the demand for them—in Fiscal Year 2020 alone, more than 665 000 individuals were on waiting lists to receive HCBS, highlighting the ongoing unmet needs for long-term services and supports.^24^ Additionally, while nursing home closures were increasing before the pandemic, closures increased sharply during the COVID-19 pandemic, and an increasing number of nursing homes experienced financial distress.^3,25,26^ Increased nursing home closures could further exacerbate access issues, particularly in counties where the only nursing facility closes, thereby creating nursing home deserts.^25^ Collectively, these trends suggest that to meet these ongoing needs, nursing homes will continue to provide critical health care services to the population requiring long-term residential care for individuals who cannot live independently in the community.^1^

The broad decline in nursing home bed supply, which intensified during the COVID-19 pandemic, may impede access to care and contribute to worsening outcomes for the growing population that relies on nursing homes for postacute care after hospitalization. Over 40% of Medicare beneficiaries receive care in nursing homes after a hospitalization,^27,28^ and postacute care received in a nursing home (or skilled nursing facility) is associated with lower rates of hospital readmission compared to postacute home health care.^27^ Moreover, within hospital catchment areas, the supply of local nursing home beds per capita is negatively associated with hospital readmissions for acute myocardial infarctions and heart failure.^29^ If access to nursing home care is not commensurate with population-level postacute care needs, older adults may be at greater risk for rehospitalization and other adverse outcomes.

Our findings also indicate that the share of 4- or 5- star beds declined. This appears to largely be driven by areas of the country with increasing nursing home bed supply where there may be a relative increase in low-quality beds. In the few areas where bed supply increased, the percentage of available low-quality beds and for-profit beds increased. Given prior evidence suggesting that for-profit nursing homes are associated with lower quality of care,^30^ areas with increasing supply of beds may not necessarily reflect an increase in supply of high-quality beds. Monitoring the improvement or worsening of newly available low-quality beds can inform targeted policies to incentivize improvements in quality of care.

Policies supporting access to high-quality nursing home care are critical to meet the needs of an aging population. To support access, the National Academies of Science, Engineering, and Medicine released a report in 2022 outlining recommendations to improve the quality of care in nursing homes.^1^ Recommendations included, but were not limited to, eliminating certificate of need laws and providing financial incentives for innovative models of care, such as The Green House Project.^1^ To ensure access to high-quality postacute care while containing costs, the Committee on the Quality of Care in Nursing Homes has recommended expanding value-based payments for postacute care to shift financial accountability of postacute care from nursing homes to hospitals.^1^ In addition, future research should focus on identifying patients for whom HCBS are truly effective substitutes for institutional care.

### Limitations

This study had limitations. First, we used the population aged 65 years or older per county per year as a proxy for nursing home demand. This is an imperfect proxy as we were unable to adjust for the number of older adults with disabilities who might be more likely to require nursing home care, or the number of individuals younger than 65 years who require nursing home care for other reasons. Second, senior housing residential bed data was only available for the 140 largest metropolitan statistical areas from 2015 to 2019. As such, we could not extrapolate our findings outside of these geographic areas. Third, we examine changes in nursing home bed star-rating by dichotomizing the 5-star rating scale to identify high- and low-quality beds and acknowledge the potential loss of information by dichotomizing a complex measure, such as quality of care.

## Conclusions

In this national, serial cross-sectional study, we found that the supply of nursing home beds has declined for most US counties and that the supply of high-quality beds has not kept pace with the demographics of an aging population. The decline in nursing home bed supply and senior housing beds collectively suggest that more work is needed to understand whether the long-term residential care needs of an aging population are being met. Understanding the supply of nursing home beds and associated geographic variation can inform targeted policies to best support older adults requiring nursing home care. Policies designed to ensure access and improve quality of nursing home care are critical to meeting the expected demand for services among older adults.

## Notes

Supplement 1. **eFigure.** Distribution of Change in Number of Beds **eTable 1.** Nursing Home Bed Characteristics Across Counties Categorized by Increasing and Decreasing of Nursing Home Beds, 2011-2020 **eTable 2.** Nursing Home Bed Characteristics Across Counties Categorized by Increasing and Decreasing of Nursing Home Beds, 2011-2019

Supplement 2.
Data Sharing Statement
