# Errors in Figure 2

**DOI:** 10.1001/jamanetworkopen.2023.11154

**Published:** 2023-04-17

In the Original Investigation titled “Trends in Supply of Nursing Home Beds, 2011-2019,”^1^ published March 1, 2023, there is an error in Panel C of Figure 2. The figure should have shown the mean number of beds adjusted per 10 000 per county by year instead of the sum of all the number of beds adjusted per 10 000 in each county by year. This article has been corrected.^1^

## References

[1] Miller KEM, Chatterjee P, Werner RM. Trends in supply of nursing home beds, 2011-2019. JAMA Netw Open. 2023;6(3):e230640. doi:10.1001/jamanetworkopen.2023.0640 36857055 PMC9978943

