# Supplementary material for: Trends in Supply of Nursing Home Beds, 2011-2019
Source: JAMA Netw Open. 2023 Mar 1;6(3):e230640. doi: 10.1001/jamanetworkopen.2023.0640 (PMC9978943; doi:10.1001/jamanetworkopen.2023.0640)
Supplement: Supplement 1. — eFigure. Distribution of Change in Number of Beds eTable 1. Nursing Home Bed Characteristics Across Counties Categorized by Increasing and Decreasing of Nursing Home Beds, 2011-2020 eTable 2. Nursing Home Bed Characteristics Across Counties Categorized by Increasing and Decreasing of Nursing Home Beds, 2011-2019 [file jamanetwopen-e230640-s001.pdf]

Supplemental Online Content

Miller KEM, Chatterjee P, Werner RM. Trends in supply of nursing home beds, 2011-2019. *JAMA Netw Open*. 2023;6(3):e230640. doi:10.1001/jamanetworkopen.2023.0640

eFigure. Distribution of Change in Number of Beds
eTable 1. Nursing Home Bed Characteristics Across Counties Categorized by Increasing and Decreasing of Nursing Home Beds, 2011-2020
eTable 2. Nursing Home Bed Characteristics Across Counties Categorized by Increasing and Decreasing of Nursing Home Beds, 2011-2019

This supplemental material has been provided by the authors to give readers additional information about their work.

© 2023 Miller KEM et al. *JAMA Network Open.*

eFigure 1. Distribution of Change in Number of Beds

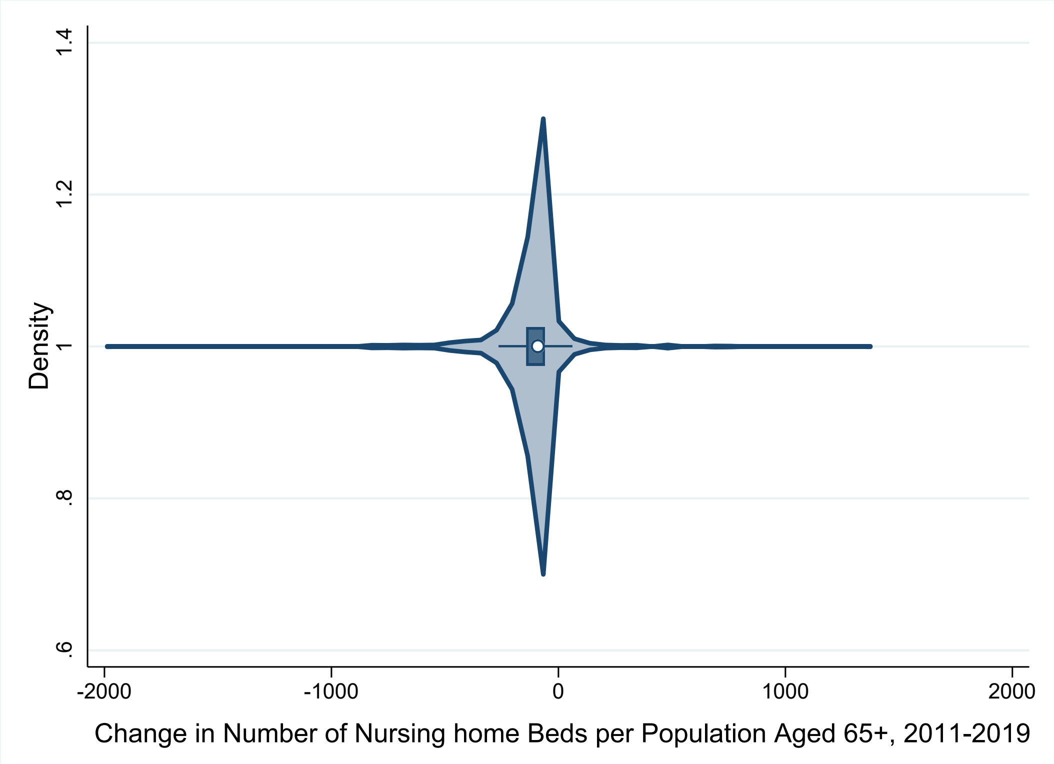

The eFigure visualizes the distribution of the change in number of nursing home beds per adults aged 65 or older from 2011-2019. The white dot represents the median; the rectangle, represents the interquartile range; and the thin horizontal line represents the remainder of the distribution excluding outliers. The shaded areas reflect the kernel density illustrating the higher/lower probability of a county adopting the given value.

© 2023 Miller KEM et al. *JAMA Network Open.*

eTable 1. Nursing Home Bed Characteristics Across Counties Categorized by Increasing and Decreasing of Nursing Home Beds, 2011-2020

**Number of Counties, n (%)**	**All counties with at least one nursing home**	**Counties with at least one nursing home in 2011 or 2020**		
		Decreasing	No Change	Increasing
	2916	N=2766 (94.9)	N=33 (1.1)	N=117 (4.0)
Number of beds per 10,000 adults 65+ per county in 2011, mean (SD)	558.7 (293.6)	555.6 (278.8)	656.3 (329.0)	603.3 (527.5)
Change in number of beds 2011 to 2020, mean (SD)	-158.4 (190.0)	-174.3 (176.7)	-0.4 (6.8)	172.7 (197.7)
Percent [a] of for-profit beds in 2011, mean (SD)	65.6 (36.8)	66.3 (36.4)	56.4 (41.2)	48.6 (44.7)
Percentage point change in for-profit beds 2011 to 2020, mean (SD)	0.2 (23.3)	0.0 (23.1)	1.7 (30.7)	4.9 (24.6)
Percent [a] of chain-affiliated beds in 2011, mean (SD)	55.1 (37.1)	55.5 (36.8)	57.2 (40.7)	44.0 (44.1)
Percentage point change in chain-affiliated beds 2011 to 2020, mean (SD)	3.2 (32.2)	3.0 (31.8)	10.8 (28.9)	4.8 (42.2)
Percent [a] of 4- or 5-star beds in 2011, mean (SD)	40.6 (36.2)	40.5 (35.9)	34.8 (36.9)	47.1 (44.6)
Percentage point change in 4- or 5-star beds 2011 to 2020, mean (SD)	2.7 (45.9)	2.9 (45.4)	-0.8 (52.4)	-1.0 (55.7)
Percent of residents whose primary support is Medicaid [b] in 2011, mean (SD)	62.8 (14.6)	63.4 (13.3)	60.0 (17.5)	48.8 (28.4)
Percentage point change in percent of residents whose primary support is Medicaid 2011 to 2020, mean (SD)	-15.8 (33.2)	-16.5 (32.5)	-12.8 (36.3)	0.5 (43.2)

Note: Calculations of nursing home beds are adjusted for 10,000 adults aged 65+ in the nursing home's county for 2011 to 2019.

[a] Percent is based on all nursing home beds in a county (e.g. the percent of for-profit nursing home beds out of all nursing home beds in a county).

[b] Percent of residents whose primary support is Medicaid is measured at the nursing home level.

© 2023 Miller KEM et al. *JAMA Network Open.*

eTable 2. Nursing Home Bed Characteristics Across Counties Categorized by Increasing and Decreasing of Nursing Home Beds, 2011-2019

**Number of Counties, n (%)**	**Counties with at least one nursing home in 2011 or 2019**		
	Decreasing	No Change	Increasing
	N=2747 (94.2%)	N=24 (0.8%)	N=145 (5.0%)
**Number of beds per county in 2011, mean (SD)**	553.5 (275.9)	0.0 (0.0)	748.7 (451.9)
**Change in number of beds 2011 to 2019, mean (SD)**	-128.6 (123.8)	385.8 (268.9)	90.2 (121.2)
**Percent* of for-profit beds in 2011, mean (SD)**	66.5 (36.2)	0.0 (0.0)	50.1 (45.1)
**Percentage point change in for-profit beds 2011 to 2019, mean (SD)**	0.2 (22.8)	72.71 (43.59)	1.4 (31.3)
**Percent* of chain-affiliated beds in 2011, mean (SD)**	55.7 (36.6)	0.0 (0.0)	43.8 (43.8)
**Percentage point change in chain-affiliated beds 2011 to 2019, mean (SD)**	1.7 (32.9)	49.74 (48.26)	3.4 (43.0)
**Percent[a] of 4- or 5-star beds in 2011, mean (SD)**	40.4 (35.8)	0.0 (0.0)	44.1 (42.8)
**Percentage point change in 4- or 5-star beds 2011 to 2019, mean (SD)**	0.0 (45.6)	45.18 (47.75)	-3.5 (53.3)
**Percent of residents whose primary support is Medicaid[b] in 2011, mean (SD)**	63.5 (13.3)	0.0 (0.0)	60.6 (15.5)
**Percentage point change in percent of residents whose primary support is Medicaid 2011 to 2019, mean (SD)**	-0.8 (14.7)	76.6 (15.3)	4.0 (12.8)

Note: Calculations of nursing home beds are adjusted for 10,000 adults aged 65+ in the nursing home's county for 2011 to 2019.

© 2023 Miller KEM et al. *JAMA Network Open.*

[a] Percent is based on all nursing home beds in a county (e.g. the percent of for-profit nursing home beds out of all nursing home beds in a county).
[b] Percent of residents whose primary support is Medicaid is measured at the nursing home level.

© 2023 Miller KEM et al. *JAMA Network Open.*
