# Supplementary material for: Trends in Supply of Nursing Home Beds, 2011-2019
Source: JAMA Netw Open. 2023 Mar 1;6(3):e230640. doi: 10.1001/jamanetworkopen.2023.0640 (PMC9978943; doi:10.1001/jamanetworkopen.2023.0640)
Supplement: Supplement 2. — Data Sharing Statement [file jamanetwopen-e230640-s002.pdf]

Data Sharing Statement

Miller. Trends in Supply of Nursing Home Beds, 2011-2019. *JAMA Netw Open*. Published March 01, 2023. doi:10.1001/jamanetworkopen.2023.0640

Data

Data available: The program and list of data sources that support the findings of this study are available in the repository "Trends in Supply of Nursing Home Beds" at https://github.com/kemmiller/Trends-in-Supply-of-Nursing-Home-Beds.
